# Preservation of ∼12-h ultradian rhythms of gene expression of mRNA and protein metabolism in the absence of canonical circadian clock

**DOI:** 10.3389/fphys.2023.1195001

**Published:** 2023-05-30

**Authors:** Bokai Zhu, Silvia Liu

**Affiliations:** ^1^ Aging Institute of UPMC, University of Pittsburgh School of Medicine, Pittsburgh, PA, United States; ^2^ Pittsburgh Liver Research Center, University of Pittsburgh, Pittsburgh, PA, United States; ^3^ Division of Endocrinology and Metabolism, Department of Medicine, University of Pittsburgh School of Medicine, Pittsburgh, PA, United States; ^4^ Department of Pathology, University of Pittsburgh School of Medicine, Pittsburgh, PA, United States

**Keywords:** ultradian and circadian rhythms, proteostasis, mRNA metabolism, X-box binding protein 1 (XBP1), *Drosophila* S2 cell

## Abstract

**Introduction:** Besides the ∼24-h circadian rhythms, ∼12-h ultradian rhythms of gene expression, metabolism and behaviors exist in animals ranging from crustaceans to mammals. Three major hypotheses were proposed on the origin and mechanisms of regulation of ∼12-h rhythms, namely, that they are not cell-autonomous and controlled by a combination of the circadian clock and environmental cues, that they are regulated by two anti-phase circadian transcription factors in a cell autonomous manner, or that they are established by a cell-autonomous ∼12-h oscillator.

**Methods:** To distinguish among these possibilities, we performed a *post hoc* analysis of two high temporal resolution transcriptome dataset in animals and cells lacking the canonical circadian clock.

**Results:** In both the liver of BMAL1 knockout mice and *Drosophila* S2 cells, we observed robust and prevalent ∼12-h rhythms of gene expression enriched in fundamental processes of mRNA and protein metabolism that show large convergence with those identified in wild-type mice liver. Bioinformatics analysis further predicted ELF1 and ATF6B as putative transcription factors regulating the ∼12-h rhythms of gene expression independently of the circadian clock in both fly and mice.

**Discussion:** These findings provide additional evidence to support the existence of an evolutionarily conserved 12-h oscillator that controls ∼12-h rhythms of gene expression of protein and mRNA metabolism in multiple species.

## Introduction

Biological rhythms are crucial for life on Earth and have provided evolutionary advantages to all living organisms, from bacteria to mammals including humans. These rhythms encompass diverse oscillators that regulate different aspects of biology, including the cell cycle that controls cell-division, infradian (with period longer than 24 h) hormone cycles that regulate the timing of organismal development, and circadian and ultradian (with period less than 24 h) oscillators that regulate organismal activity, physiology, metabolism, and cellular activity (Laje et al., 2018; Gliech and Holland, 2020; Mofatteh et al., 2021).

The circadian clock regulates daily cycles in many organisms and is based on a molecular transcriptional-translational feedback loop (TTFL) involving core circadian clock genes of BMAL1, CLOCK, PER and CRY in mammals (Takahashi, 2017). The TTFL is responsible for establishing and maintaining cell-autonomous 24-h rhythms of cellular gene expression, metabolic processes, and organismal behavior (Asher and Schibler, 2011; Roenneberg and Merrow, 2016; Takahashi, 2017). In addition to 24-h circadian rhythms, biological rhythms have also been shown to have ‘harmonics’ - rhythms with frequencies that are positive integer multiplier of 1.15*10e-5 Hz (24 h period), such as those that cycle with periods close to 12-, 8- or 4-h (Zhu et al., 2018; Kembro et al., 2023). Oscillations cycling at harmonics frequencies in mammals were first comprehensively characterized in mouse liver using high-resolution temporal microarray in 2009 by Hughes et al. (2009). Analysis of these data with both Fisher’s G and COSOPT methods yielded a few hundred genes cycling with an approximately 12-h period (Hughes et al., 2009). Since then, several studies have validated this initial discovery and further expanded the repertoire of ∼12-h mouse hepatic transcriptome to thousands of genes (van der Veen and Gerkema, 2017; Zhu et al., 2017). Strongly contributing to this expansion is the development of novel analytical methods capable of unmasking ultradian from superimposed/co-expressed circadian oscillations (van der Veen and Gerkema, 2017; Ananthasubramaniam et al., 2018; Antoulas et al., 2018; van der Veen et al., 2022). In addition to liver, wide prevalence of ∼12-h rhythms of gene expression were further identified in murine white adipose tissue, brown adipose tissue, adrenal gland, aorta, muscle and cornea using multiple mathematical/statistical methods (van der Veen and Gerkema, 2017; Zhu et al., 2017; Antoulas et al., 2018; Castellana et al., 2018; Zhu et al., 2018; El-Athman et al., 2019; Genov et al., 2019; Jiao et al., 2019; Meng et al., 2020; Pan et al., 2020; Zhu, 2020; Ballance and Zhu, 2021; Asher and Zhu, 2022; Dion et al., 2022; Meng et al., 2022). Using spectrum analytic methods including Fourier transform, wavelet, autocorrelation analysis and eigenvalue/pencil, ∼12-h rhythms of locomotor activity, energy homeostasis and/or feeding rhythms were also uncovered from rodents (Zhu et al., 2017; Hasanpour et al., 2021; Kembro et al., 2023).

Gene Ontology (GO) analysis of ∼12-h transcriptome in different mouse tissues revealed strong enrichment in the entire central dogma information flow (CEDIF) process, ranging from transcription initiation, mRNA processing and export, ribosome biogenesis, translation initiation to protein folding, degradation, processing and sorting in the endoplasmic reticulum (ER) and Golgi and include both anabolic and catabolic processes (Meng et al., 2020; Pan et al., 2020; Zhu, 2020). More importantly, in a most recent endeavor, using time of death information as a surrogate for circadian time, ∼12-h rhythms of gene expression were identified in the dorsolateral prefrontal cortex regions of normal human subjects (Scott et al., 2023). Intriguingly, human subjects with schizophrenia lose ∼12-h rhythms in genes associated with unfolded protein response (UPR) and neuronal structural maintenance in the same brain regions (Scott et al., 2023).

While the evidence supporting the existence of ∼12-h rhythms in mammals is compelling, the origin and mechanisms of regulation of these rhythms are poorly understood and have been the subject of several theories over the past decade. One of the early hypotheses suggested that these ∼12-h rhythms are not cell-autonomous and instead established by the combined effects of circadian clock and fasting-feeding cues (Hughes et al., 2009; Cretenet et al., 2010). This hypothesis was mainly supported by the observation that several ∼12-h rhythms of proteostasis gene expression were disrupted in *Cry1/Cry2* double knockout mice and/or during altered feeding regimen in wild-type mice (Cretenet et al., 2010). Alternatively, it was suggested that two circadian transcription factors with anti-phasic transcriptional activity and binding cooperativity are theoretically capable of establishing 12-h rhythms of gene expression in a cell-autonomous manner (Westermark and Herzel, 2013). However, our recent work challenged these two paradigms, and instead proposed the existence of a dedicated and cell-autonomous 12-h oscillator responsible for the establishment and maintenance of a large proportion of ∼12-h ultradian rhythms (Zhu et al., 2017; Meng et al., 2020; Pan et al., 2020; Dion et al., 2022; Meng et al., 2022). At the center of the 12-h oscillator is spliced form of XBP1 (XBP1s), a transcription factor (TF) previously characterized as one of the master regulators for UPR. In XBP1 liver-specific knockout (XBP1 LKO) mice, ∼85% of the ∼12-h hepatic transcriptome was either abolished (54%) or dampened (31%) compared to wild-type mice, while the core circadian clock genes and most circadian clock-controlled output genes (66%) remained robustly oscillatory with a ∼24-h period (Pan et al., 2020). Further, cell-autonomous ∼12-h rhythms of *XBP1s* and several representative proteostasis gene expression were observed in synchronized mouse embryonic fibroblasts (MEFs) with or without *Bmal1* knocking down (Zhu et al., 2017; Pan et al., 2020).

While these findings support the existence of an XBP1s-dependent 12-h-oscillator, it remains largely undetermined to what extent these ∼12-h rhythms of gene expression are preserved in the absence of circadian clock *in vivo* and *in vitro*. In our prior study, while we did observe that many of these ∼12-h rhythms of gene expression are visibly intact in the liver of BMAL1 knockout (Yang et al., 2016) and CLOCK ^Δ19^ mutant (Miller et al., 2007) mice (Zhu et al., 2017), the short duration (24 h) of these two temporal transcriptome datasets prevented us from rigorous and unbiased identification of all ∼12-h transcriptome resistant to circadian clock ablation. Herein, utilizing a recently published high temporal resolution/duration (4-h interval for a total of 48 h with quadruplicates at each time point) hepatic RNA-seq dataset from whole body BMAL1 knockout mice kept under constant darkness (Aviram et al., 2022), we uncovered widespread hepatic ∼12-h rhythms of gene expression implicated in mRNA and protein metabolism. We further corroborated our findings with an additional cellular model, the *Drosophila* S2 cells which do not express canonical circadian clock genes (Rey et al., 2018), and identified hundreds of ∼12-h transcripts involved in protein and mRNA metabolic pathways. Our findings thus provide further support for the existence of an evolutionarily conserved 12-h oscillator regulating ∼12-h rhythms of genetic information flow.

## Result

### ∼12-h ultradian rhythms of gene expression are prevalent in the liver of free-running BMAL1 knockout mice

To investigate the extent to which ∼12-h rhythms of gene expression are present and functionally preserved in the absence of the circadian clock *in vivo*, we performed a *post hoc* analysis of a recently published temporal RNA-seq data collected from the liver of male whole body BMAL1 knockout mice (3 months of age) (Bunger et al., 2000) that were kept under constant darkness after being entrained under 12-h/12-h light/dark cycle for 14 days (Aviram et al., 2022). This specific dataset was selected due to the following reasons. First, *Bmal1* is the only gene that when knocked out in mice leads to a complete abolishment of circadian locomotor and feeding cycles, and as a result BMAL1 knockout mice is a widely-accepted model for circadian clock ablation (Bunger et al., 2000). Secondly, in this study, BMAL1 knockout mice were kept in constant darkness, thus ruling out the possibility that any ultradian rhythms hereby uncovered are driven by light cues. Thirdly, of all the available temporal transcriptome dataset from BMAL1 knockout mice, it has the best temporal resolution (at 4-h interval), duration (for a total of 48 h) and sample size (n of four), thus permitting rigorous testing for ∼12-h ultradian rhythms.

**FIGURE 1:**
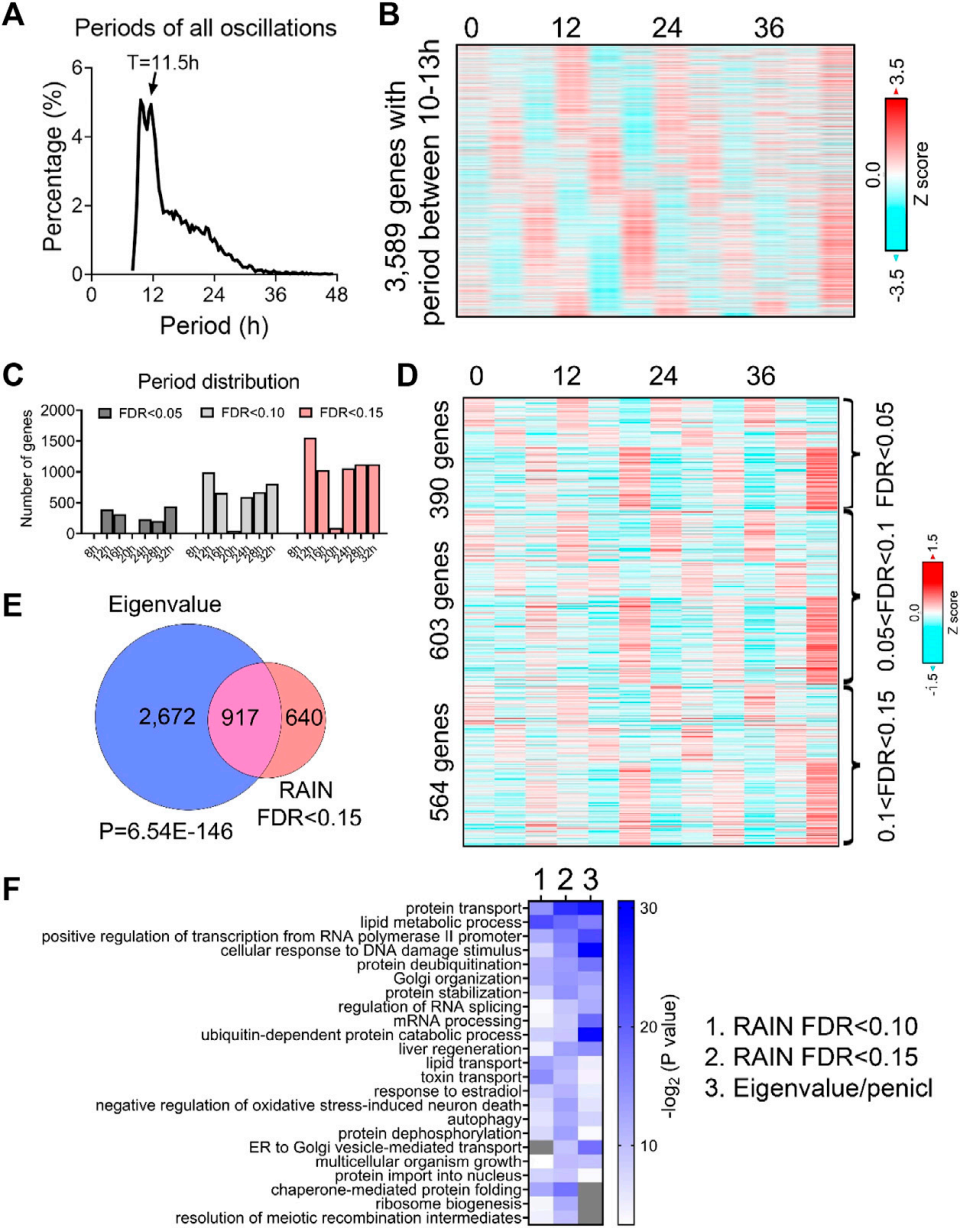
Prevalent ∼12-h rhythms of gene expression in the liver of BMAL1 knockout mice. **(A)** Distribution of periods uncovered from all oscillations by the eigenvalue/pencil method. **(B)** Heatmap of ∼12-h rhythms of gene expression uncovered by the eigenvalue/pencil method. **(C)** Number of genes cycling with different periods uncovered by the RAIN method, with different FDR cut-off. **(D)** Heatmap of ∼12-h rhythms of gene expression ranked by different FDR cut-off. **(E)** Venn diagram comparing ∼12-h transcriptome uncovered by the eigenvalue/pencil and RAIN (FDR cut-off of 0.15) method. *p*-value calculated by Chi-square test. **(F)** GO analysis showing enriched biological pathways of ∼12-h genes revealed by different methods using all mice genes as background.

To unbiasedly reveal all oscillations from BMAL1 knockout mice liver, we initially applied the eigenvalue/pencil method (Zhu et al., 2017; Antoulas et al., 2018), which is a nonparametric spectrum analytical algorithm that deconvolutes raw temporal data into a linear combination of exponentials (sinusoidal waveforms with a decay factor) plus noise. Consistent with the role of BMAL1 as a master regulator of circadian rhythms, the prevailing population of circadian oscillations observed in wild-type mice are lost in the absence of BMAL1 (Figure 1A). By contrast, 3,589 genes cycling with periods between 10–13 h were identified (Figures 1A, B; Supplementary Table S1). Since the eigenvalue/pencil algorithm is a non-statistical signal processing method, we used a permutation-based method that randomly shuffles the time label of gene expression data to determine the false discovery rate (FDR) for the ∼12-h genes (Rey et al., 2018; Pan et al., 2020). This way, the FDR for the ∼12-h genes was estimated to be 0.31, a range in line with prior studies of circadian rhythms (Mauvoisin et al., 2014; Robles et al., 2014; Rey et al., 2018). Furthermore, the calculated FDR is likely an overestimation since amplitude and phase information for the ∼12-h rhythms is not considered in the permutation dataset.

To assess the robustness of the uncovered ∼12-h oscillations in BMAL1 knockout mice, we applied an orthogonal rhythm-identification algorithm, RAIN (Thaben and Westermark, 2014), which detects rhythms with arbitrary waveforms exhibiting a single pre-specified period and has been successfully used to uncover ∼12-h ultradian rhythms from high temporal resolution dataset in prior studies (Meng et al., 2020; Pan et al., 2020). In line with the eigenvalue/pencil method, 12-h were also the dominant oscillations identified by the RAIN method, with 390, 993 and 1,557 12-h genes identified with FDR (or q value) cut-off of 0.05, 0.1 and 0.15, respectively (Figures 1C, D; Supplementary Figures S1A–C; Supplementary Table S2). Importantly, the robustness of 12-h oscillations for genes under different FDR cut-off does not look visually different (Figure 1D; Supplementary Figures S1A, B), and ∼12-h genes identified by both methods further showed large convergence (Figure 1E).

We subsequently performed GO analysis on ∼12-h genes identified by both methods using all mouse genes or all hepatically expressed genes as background, and identified commonly enriched pathways of lipid metabolism, proteostasis (including ribosome assembly, protein transport, protein processing, protein folding/stability/degradation, autophagy, and Golgi organization), and to a lesser degree, mRNA metabolism (transcription, RNA splicing and processing) (Figure 1F; Supplementary Figure S1D). Among these pathways, protein and mRNA metabolism were previously observed to be highly enriched in hepatic ∼12-h transcriptome in wild-type mice as well (Meng et al., 2020; Pan et al., 2020). Collectively, these results indicate the presence of prevalent ∼12-h ultradian rhythms of gene expression in the absence of the circadian clock. Furthermore, since the locomotor activity and feeding behaviors of BMAL1 knockout mice are completely arrhythmic under constant darkness condition (Bunger et al., 2000), it suggests that these ∼12-h rhythms of gene expression are highly robust and resistant to feeding cues.

### Hepatic ∼12-h rhythms of gene expression and functionality are conserved between wild-type and BMAL1 knockout mice under free-running condition

We next aim to determine the extent to which ∼12-h transcriptome is conserved between wild-type and BMAL1 knockout mice. Those commonly found in both genotypes are likely controlled by the 12-h oscillator, while “*de novo*” ∼12-h genes only observed in BMAL1 knockout mice may reflect compensatory gain of new ultradian rhythms secondary to circadian clock ablation. We selected the hepatic RNA-seq dataset published by Pan et al. (2020) for comparison due to the two following reasons. First of all, this dataset has the highest temporal resolution (at 2-h interval), duration (48 h) and sample size (duplicates at each time point) among all temporal hepatic RNA-seq dataset for wild-type mice kept under constant darkness condition (Brooks et al., 2022). Secondly, the strain (C57BL/6), sex (male) and age (8–12 weeks) of these mice are comparable to those of BMAL1 knockout mice.

**FIGURE 2:**
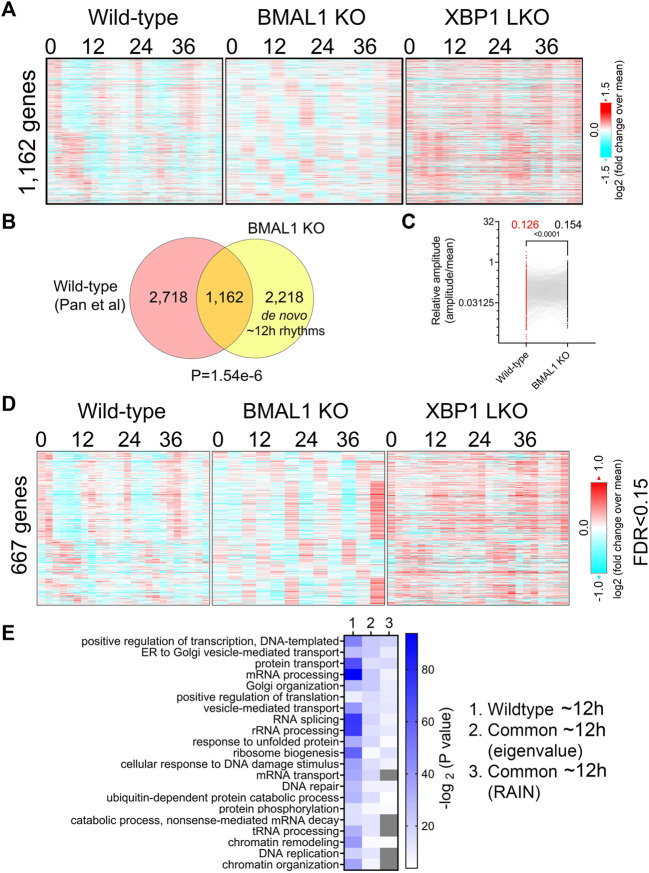
Preservation of ∼12-h gene program and functionality between wild-type and BMAL1 knockout mice. **(A)** Heatmap of 1,162 commonly found ∼12-h rhythm gene expression in wild-type, BMAL1 knockout and XBP1 LKO mice. **(B)** Venn diagram comparing ∼12-h rhythm genes in wild-type and BMAL1 knockout mice. *p*-value is calculated by Chi-square test. **(C)** Relative amplitude of ∼12-h oscillations for 1,162 genes in wild-type and BMAL1 knockout mice. **(D)** Heatmap of 667 commonly found ∼12-h rhythm gene expression in wild-type, BMAL1 knockout and XBP1 LKO mice (FDR cut-off of 0.15). **(E)** GO analysis showing enriched biological pathways of ∼12-h genes found in wild-type or in both wild-type and BMAL1 knockout mice using all mice genes as background.

Comparing ∼12-h genes uncovered by the eigenvalue/pencil method in both datasets revealed a statistically significant (*p*-value of 1.54e-6) overlap of 1,162 commonly shared genes, indicating preservation of ∼12-h gene programs between wild-type and BMAL1 knockout mice (Figures 2A, B; Supplementary Figure S2A). The relative amplitude (absolute amplitude normalized to average gene expression) of shared ∼12-h genes is comparable between the two genotypes, albeit with a slight (but statistically significant) increase observed in BMAL1 knockout mice (Figure 2C). GO terms (regardless of the background gene sets used) associated with those 1,162 commonly shared ∼12-h genes are highly similar to those of ∼12-h genes in wild-type mice, namely, proteostasis (protein transport, Golgi organization, translation regulation, UPR, tRNA processing, and ubiquitin-mediated protein degradation) and mRNA metabolism (transcription regulation, mRNA processing and splicing and mRNA transport). The convergence of ∼12-h genes and their functionality between wild-type and BMAL1 knockout mice are further validated with the RAIN method (FDR cut-off of 0.15) (Figures 2D, E; Supplementary Figures S2A, B).

In addition to commonly shared ∼12-h genes, many genes with ∼12-h rhythms exclusively identified in either wild-type or BMAL1 knockout mice were present (Figure 2B). A close examination of their period change in the opposite genotype reveals that 2,718 ∼12-h genes only identified in wild-type mice often exhibit periods slightly lower than 10-h in BMAL1 knockout mice, thus being labelled as not having ∼12-h oscillations (Supplementary Figure S3A). These genes are also enriched in protein and mRNA metabolic pathway as expected regardless of the background gene sets used (Supplementary Figures S3B, C). On the contrary, many of the 2,218 ∼12-h genes exclusively observed in BMAL1 knockout mice exhibit periods in the circadian range in wild-type mice and these genes are strongly enriched in lipid metabolism, mitosis, complement activation and Notch signaling, pathways that are not strongly associated with ∼12-h transcriptome in wild-type mice (Supplementary Figures S3A–C). The latter group can be categorized as *de novo* ∼12-h genes that are gained in BMAL1 knockout mice secondary to the ablation of circadian clock. Taken together, these findings provide additional evidence to support the existence of a 12-h oscillator that controls ∼12-h rhythms of gene expression of protein and mRNA metabolism in mice liver.

### Many hepatic genes are under dual circadian clock and 12-h oscillator control

**FIGURE 3:**
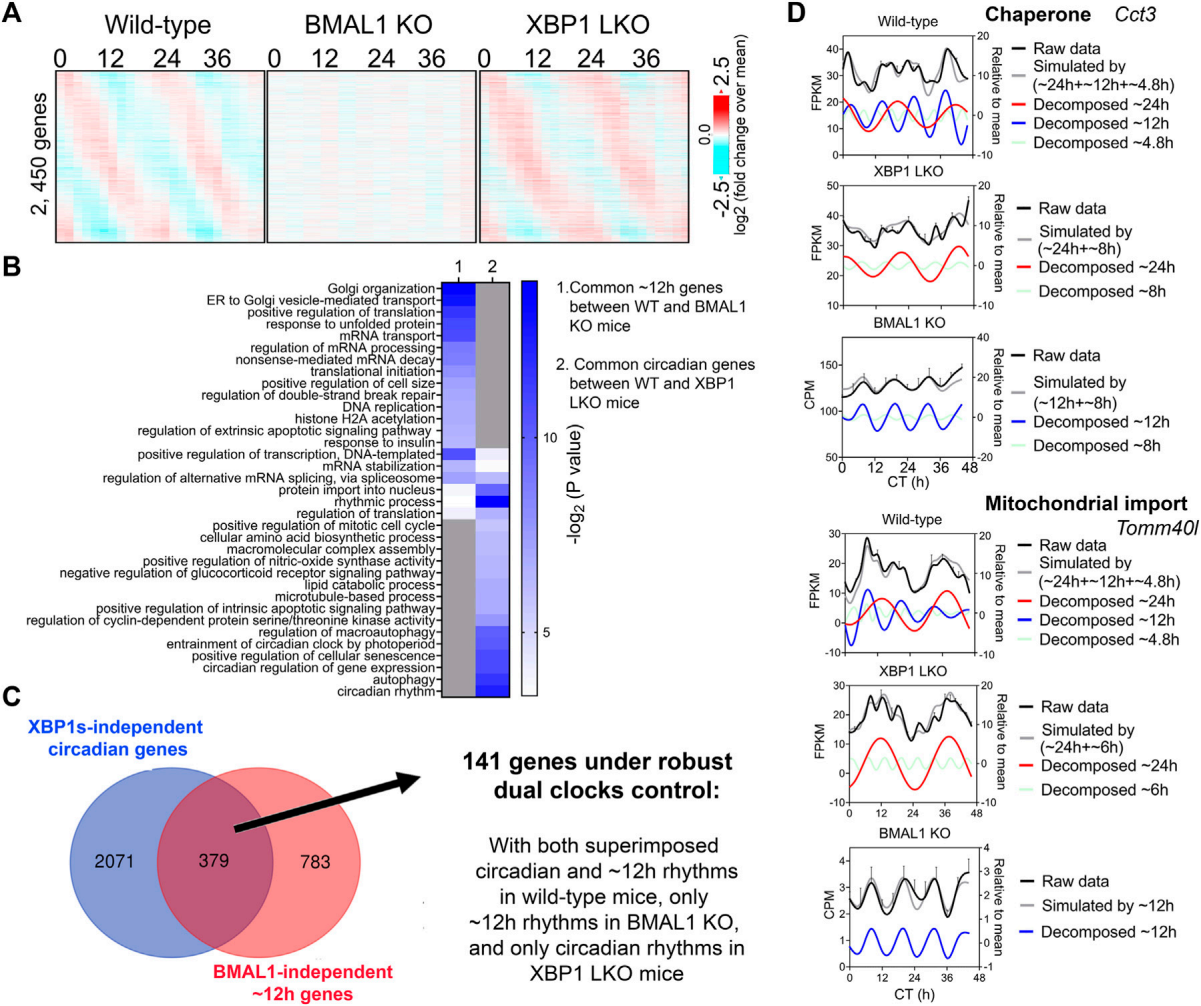
Many hepatic genes are under dual circadian clock and 12-h oscillator control. **(A)** Heatmap of 2,450 commonly found circadian gene expression (common between wild-type and XBP1 LKO mice) in wild-type, BMAL1 knockout and XBP1 LKO mice. **(B)** GO analysis showing enriched biological pathways of common ∼12-h (between wild-type and BMAL1 knockout mice) and circadian (between wild-type and XBP1 LKO mice) using hepatically expressed genes as background. **(C)** Venn diagram illustrating a total of 141 genes under robust dual clock control. **(D)** Eigenvalue/pencil deconvolution of *Cct3* and *Tomm40l* temporal gene expression profiles in wild-type, BMAL1 knockout and XBP1 LKO mice. Gray line in each graph illustrates simulated temporal gene expression profile by addition of all superimposed oscillations.

∼12-h genes commonly identified in both wild-type and BMAL1 knockout mice are likely under 12-h oscillator control. In agreement with this hypothesis, their ∼12-h rhythms of gene expression are impaired in XBP1 (LKO) mice (Pan et al., 2020) (Figures 2A, D). Conversely, as previously reported, core circadian clock and the majority of circadian output genes are maintained in XBP1 LKO mice (Meng et al., 2020; Pan et al., 2020), and abolished in BMAL1 knockout mice, as expected (Figure 3A). While the repertoire and functionality of circadian and ∼12-h genes in wild-type mice are largely separate (Figures 3B, C; Supplementary Figure S4A), we did observe hundreds of genes having both circadian and ∼12-h rhythms superimposed (Figure 3C). The phenomenon of superimposition of circadian and ultradian rhythms in the same genes are widespread and have been previously reported in many different organisms by other groups (Ananthasubramaniam et al., 2018; Yang et al., 2022; Psomas et al., 2023). Using a very stringent criterion where the superimposed circadian and ∼12-h rhythms need to be abolished in BMAL1 KO and XBP1 LKO mice, respectively, but maintained in XBP1 LKO and BMAL1 KO mice, respectively, we identified 141 genes under robust dual control of BMAL1-dependent circadian clock and XBP1s-dependent 12-h oscillator (Figure 3C; Supplementary Table S3). Examples of such genes include chaperone *Cct3*, mitochondria import receptor *Tomm40l* (Tom40 b), master transcriptional regulator of fatty acid oxidation *Ppara*, and RNA helicase *Ddx1* (Figure 3D; Supplementary Figure S4B). In all cases, eigenvalue/pencil method revealed coexistence/superimposition of both circadian and ∼12-h rhythms (sometimes with additional ∼4.8-h or ∼8-h rhythms as well) in wild-type mice, and more importantly, the linear addition of these rhythms closely matches the raw temporal gene expression profiles, indicating the robustness of deconvolution (Figure 3D; Supplementary Figure S4B). Interestingly, while in BMAL1 KO and XBP1 LKO mice, circadian and ∼12-h rhythms are no longer identified by the eigenvalue/pencil method, respectively, ultradian rhythms with even smaller periods (4.8∼8-h) are often observed (Figure 3D; Supplementary Figure S4B), indicating the likely existence of additional oscillators for high frequency rhythms.

### ELF1, KLF7, and ATF6B are novel putative transcription regulators of the hepatic 12-h oscillator

**FIGURE 4:**
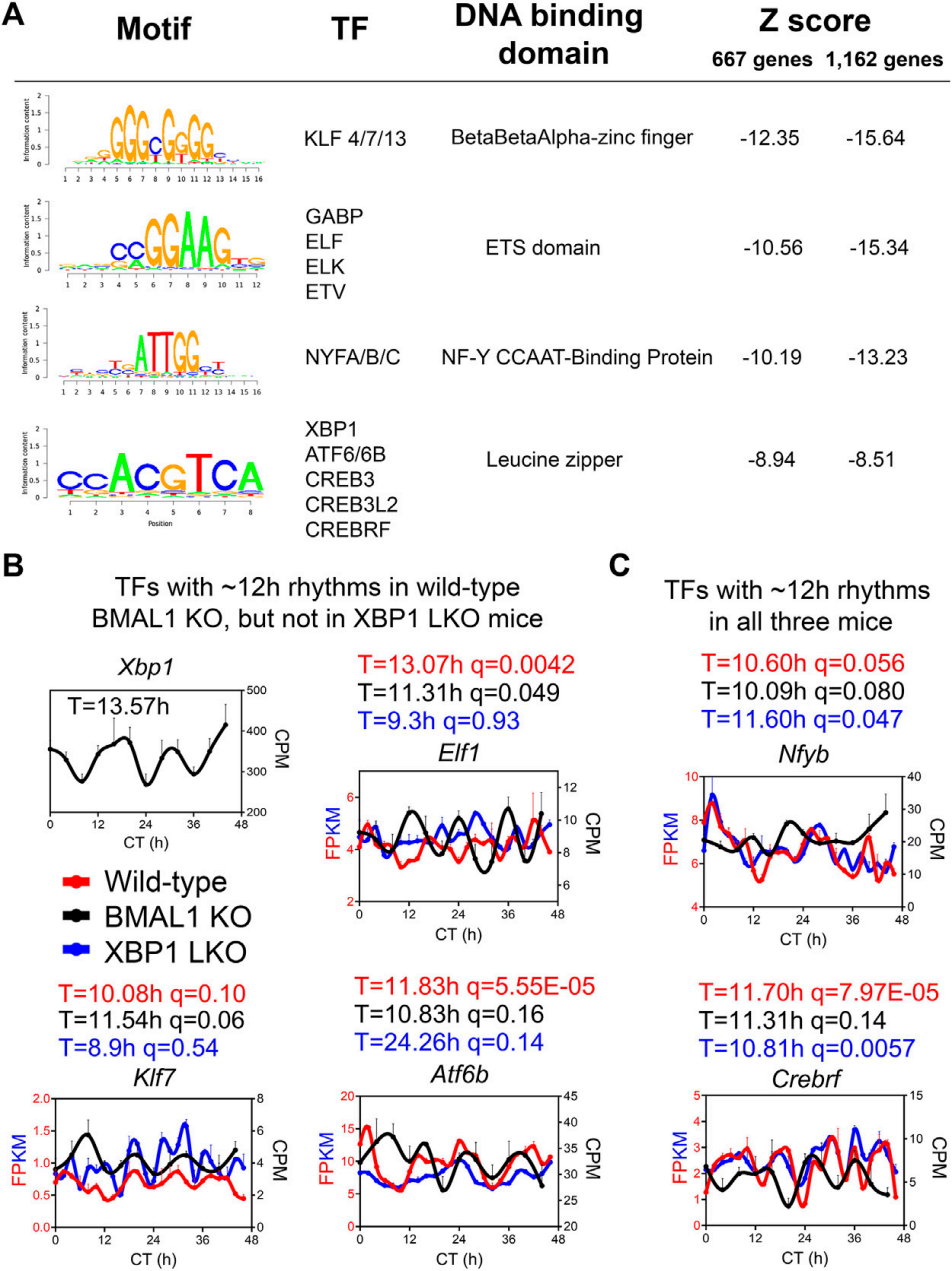
ELF1, KLF7 and ATF6B are putative transcription regulators of 12-h oscillator. **(A)** Motif analysis of promoter regions of common ∼12-h rhythm genes identified in wild-type and BMAL1 knockout mice by either method. **(B,C)** Expression of different transcription factors in wild-type, BMAL1 knockout and XBP1 LKO mice. Period identified by the eigenvalue/pencil method, and q value (FDR) for having 12-h rhythms of gene expression via the RAIN method were shown for each gene in each genotype.

To infer the gene regulatory network governing ∼12-h oscillator, we performed motif analysis on the commonly shared ∼12-h genes between wild-type and BMAL1 knockout mice identified by either the eigenvalue/pencil or RAIN methods. Regardless of the method used, we identified top motifs associated with KLF, ETS, NFY and basic leucine zipper (including ATF6, XBP1, CREB3, CREB3L2, and CREBRF) families of transcription factors (Figure 4A), consistent with previous results (Pan et al., 2020; Zhu, 2020). *XBP1* itself exhibited a ∼13-h oscillation in the liver of BMAL1 knockout mice (Figure 4B). We further cross-referenced enriched motifs with transcription factors exhibiting ∼12-h rhythms in both wild-type and BMAL1 knockout mice, but not in XBP1 LKO mice, and identified KLF7, ELF1 and ATF6B as strong putative transcriptional regulators of 12-h oscillator. Interestingly, *KLF7* was previously shown to also exhibit a ∼12-h rhythm of expression in human dorsolateral prefrontal cortex (Scott et al., 2023). In addition, we further identified NYFB and CREBRF as two transcriptional factors exhibiting ∼12-h rhythms in all three genotypes of mice (Figure 4C). Of these transcription factors, hepatic XBP1s, NFYA, NFYC and GABPB1 expressions further exhibit ∼12-h rhythms at the protein level in wild-type mice, as previously measured (Wang et al., 2018) and quantified (Dion et al., 2022), while the expression levels of GABPA/B2, ATF6/6B, NFYB, KLF7 and ELF1 were not reported from the same study.

To ensure that our analysis is robust to datasets used, we further compared the ∼12-h hepatic transcriptome of BMAL1 knockout mice with an additional wild-type dataset published by the same group (Aviram et al., 2022). The relatively low resolution (4-h interval for 48-h under constant darkness) renders this dataset not ideal for ultradian rhythms detection by statistical methods like RAIN due to the superimposition of strong circadian rhythms for many genes. Nonetheless, using eigenvalue/pencil method, we uncovered hundreds of overlapping ∼12-h transcriptome between wild-type and BMAL1 knockout mice that has 1) similar amplitude but distinct phases, 2) strong enrichment in proteostasis pathways, and 3) over-representation of KLF, bZIP, ETS or NYF TFs motifs in their promoters (Supplementary Figures S5A–H), all consistent with the results described above. Collectively, our analysis of ∼12-h rhythms in wild-type, BMAL1 knockout and XBP1 LKO mice provided novel mechanistic insights on the regulation of mammalian 12-h oscillator.

### Cell-autonomous ∼12-h rhythms of gene expression involved in mRNA and protein homeostasis are present in *Drosophila* S2 cells that lack canonical circadian clock gene expression

Having established the preservation of ∼12-h rhythms of gene expression in the absence of circadian clock in mice, we wanted to further corroborate this finding in additional animals that also lack a canonical circadian clock. We focused upon the fruit fly *Drosophila melanogaster*, where ∼12-h rhythms, to the best of our knowledge, have never been reported before. In *Drosophila melanogaster*, the model of the circadian clock regulation involves transcription factors CYCLE (CYC) and CLOCK (CLK), the homologs of BMAL1 and CLOCK in mammals, which in turn control the transcription of several repressive clock genes including *period* (*per*) and *timeless* (*tim*) (Panda et al., 2002). Besides a subset of cells located in the principal pacemaker driving daily activity rhythms and physiology, most other cells including *Drosophila* Schneider 2 (S2) cells [originally derived from a primary culture of late-stage embryos (Schneider, 1972)] do not express these core circadian clock genes and therefore do not have the necessary apparatus to form a TTFL to drive circadian oscillations (Saez and Young, 1996; Darlington et al., 1998; Rey et al., 2018). We thus deduce that if ∼12-h rhythms of gene expression with functionality like those identified in mice can be observed in S2 cells, it will provide additional evidence for the existence of a cell-autonomous 12-h oscillator independent from the canonical circadian clock.

**FIGURE 5:**
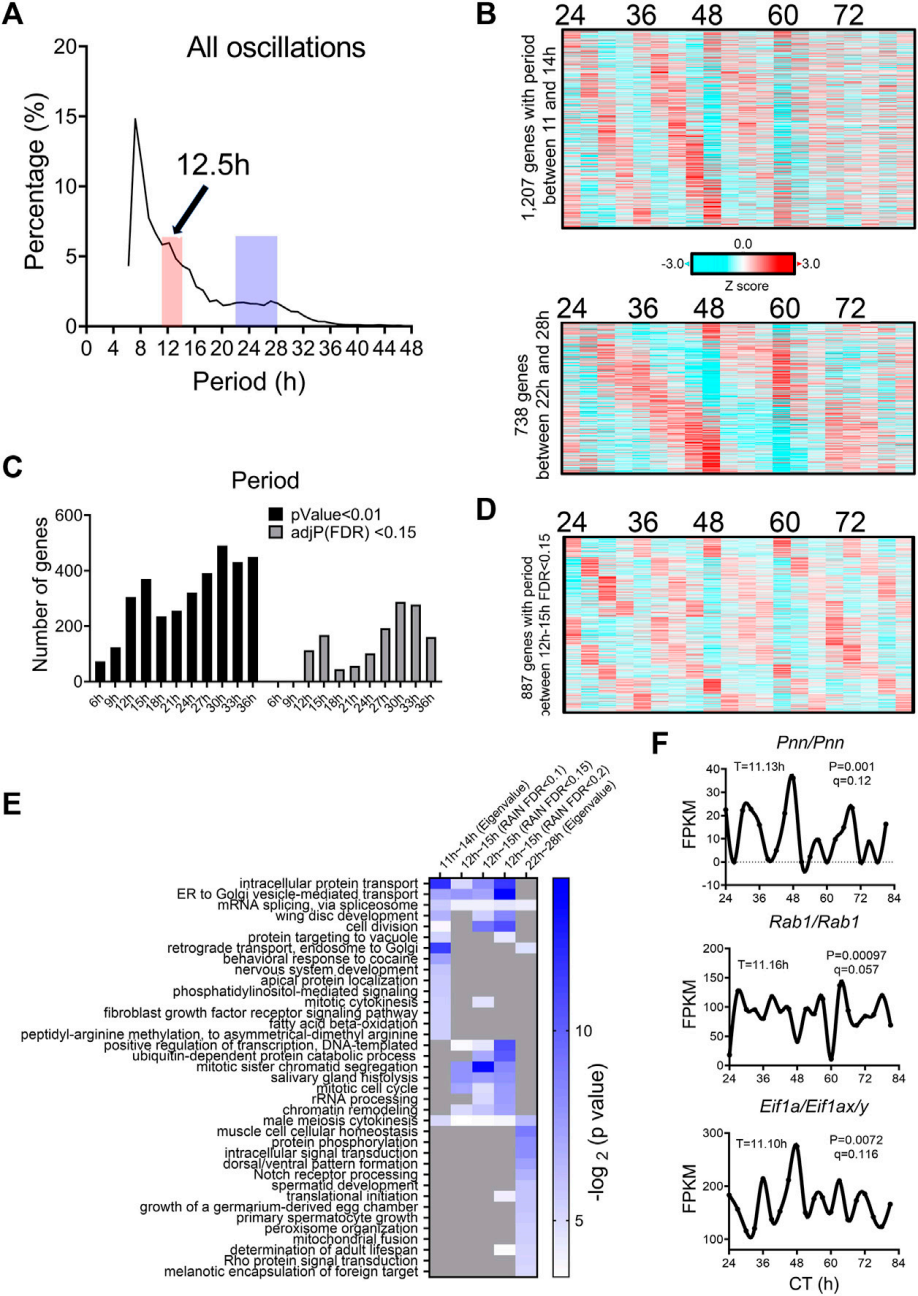
∼12-h rhythms of gene expression are present in S2 cells. **(A)** Distribution of periods uncovered from all oscillations by the eigenvalue/pencil method. **(B)** Heatmap of ∼12-h (top) and circadian rhythms (bottom) of gene expression uncovered by the eigenvalue/pencil method. **(C)** Number of genes cycling with different periods uncovered by the RAIN method, by *p*-value or FDR cut-off. **(D)** Heatmap of ∼12-h rhythms of gene expression with FDR cut-off of 0.15 via RAIN. **(E)** GO analysis of ∼12-h and circadian rhythms uncovered by different methods/FDR cut-off in S2 cells using all fly genes as background. **(F)** Representative expression of selective genes. Period identified by the eigenvalue/pencil method, and q value (FDR) for having ∼12-h rhythms of gene expression via the RAIN method were shown for each gene.

**FIGURE 6:**
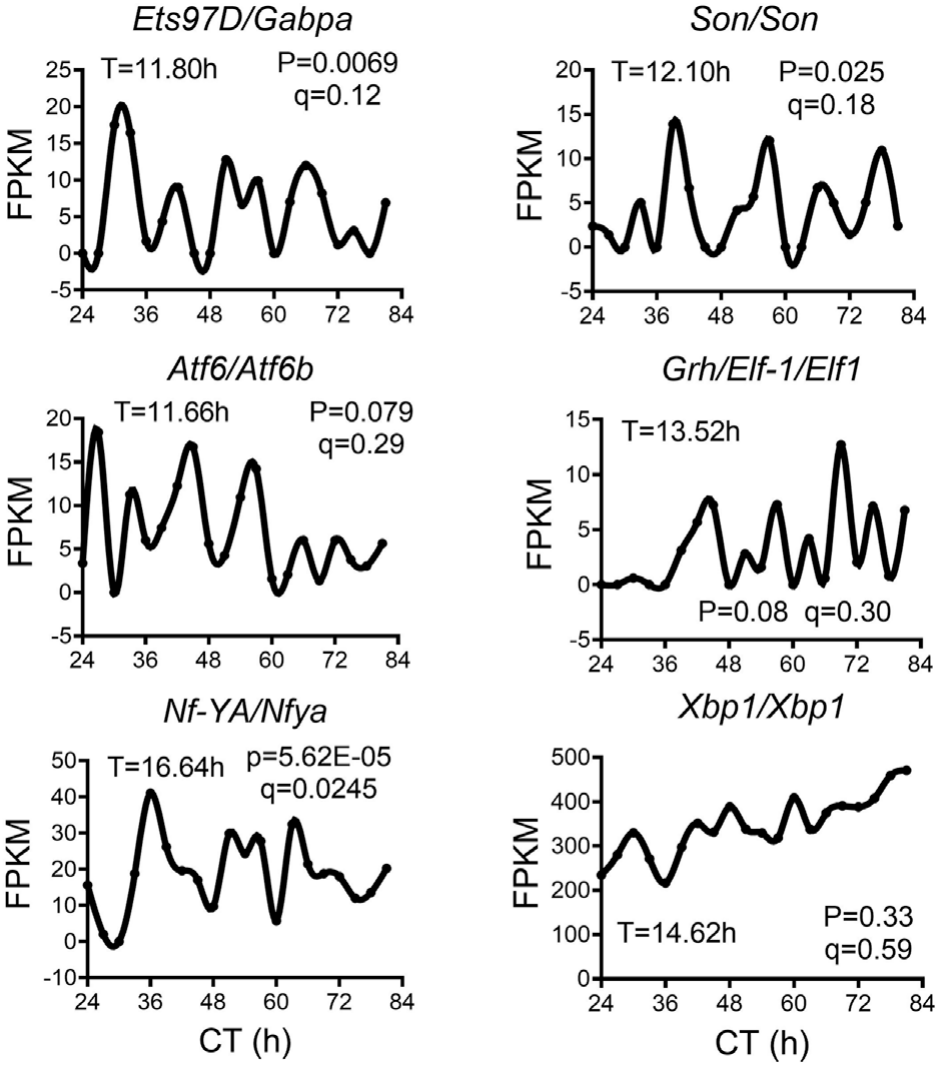
∼12-h rhythms of transcription regulators in S2 cell. Representative expression of selective transcription regulators in S2 cell. Period identified by the eigenvalue/pencil method, and q value (FDR) for having ∼12-h rhythms of gene expression via the RAIN method were shown for each gene.

We analyzed a recently published temporal RNA-seq data (Rey et al., 2018) collected from temperature entrained S2 cells (2 days of temperature cycles with 12-h at 28°C, 12-h at 23°C before free-running at 28°C) at 3-h interval for a total duration of 60 h. Using both eigenvalue/pencil and RAIN methods, we identified tens of hundreds of transcripts cycling with an ultradian period between 11 and 15 h (Figures 5A–D; Supplementary Tables S4, S5). This period range is longer than what was observed in mice, likely due to the likewise longer circadian period (22–28 h) identified in S2 cells (Figures 5A–C). This way, both ∼12 and ∼24-h rhythms are still cycling at harmonics frequencies with each other in S2 cells. GO analysis of these ∼12-h ultradian genes revealed top enriched pathways of protein transport (such as *Rab1*) and mRNA splicing (such as *Pnn*), distinct from those associated with circadian rhythms but similar to those associated with ∼12-h genes in mice (Figures 5E, F; Supplementary Figure S6). Among the putative/confirmed transcription regulators governing ∼12-h rhythms in mice, we found the fly orthologs of *Gabpa*, *Son*, *Atf6b* and *Elf1* exhibiting a period between 11 and 14 h in S2 cells (Figure 6). The fly orthologs of *Nfya* and *Xbp1* exhibit a longer period of 15–16 h in S2 cells (Figure 6). In sum, we herein provided additional evidence supporting the existence of ∼12-h rhythms of gene expression of mRNA and protein metabolism in clock-less fly cells. More importantly, the fact that ∼12-h rhythms of gene expression implicated in protein and mRNA metabolism have been found in divergent species of sea anemone (Sorek et al., 2018), *C. elegans* (van der Linden et al., 2010), fly, mice and human (Scott et al., 2023) argue for the strong evolutionary conservation of the ∼12-h oscillator.

## Discussion

In this study, by using two different analytical methods eigenvalue/pencil and RAIN, we uncovered prevalent ∼12-h rhythms of gene expression in two different models of circadian clock deficiency: BMAL1 knockout mice and *Drosophila* S2 cells. In both models, the ∼12-h gene programs are strongly enriched in protein and mRNA metabolism, consist with the gene signature previously identified for ∼12-h/circatidal genes from wild-type mice, sea anemone and even *C. elegans* (Ballance and Zhu, 2021). These findings thus lend further support for the 12-h oscillator hypothesis on the origin and regulation of ∼12-h ultradian rhythms.

In an early study Cretenet et al. (2010) reported that in the liver of CRY1/CRY2 double knockout mice, ∼12-h rhythms of expression of several UPR genes (including *Xbp1* itself) were disrupted. We believe that at least three factors can account for the discrepancies observed between BMAL1 knockout and CRY1/CRY2 knockout mice. First of all, due to the limit of technologies available at the time, the complete hepatic temporal transcriptome of CRY1/CRY2 knockout mice were not profiled. Thus, there remains the possibility that many ∼12-h rhythms of gene expression are also maintained in the liver of CRY1/CRY2 knockout mice, and future high temporal resolution profiling from these mice is needed to answer this question. Secondly, since the 12-h rhythms are sensitive to heightened metabolic and ER stress (Cretenet et al., 2010; Zhu et al., 2017; Pan et al., 2020), it is highly likely that the observed changes of 12-h rhythms in Cry1/Cry2 knockout mice is a consequence of the metabolic deficits observed in these mice, rather than due to the absence of a functional circadian clock *per se*. In fact, as reported in this study and stated by the authors (Cretenet et al., 2010), CRY1/CRY2 knockout mice liver exhibited a much higher level of triglycerides, which in turn can lead to chronic ER stress, and subsequently constitutive action of IRE1α/XBP1s signaling and impairment of ∼12-h ultradian rhythms of gene expression of proteostasis. By contrast, BMAL1 knockout mice has a normal level of hepatic triglyceride and even increased insulin sensitivity compared to wild-type mice (Jouffe et al., 2022). As a result, hepatic ∼12-h rhythms of gene expression are much better maintained in BMAL1 knockout mice than CRY1/CRY2 knockout mice. Finally, CRY proteins may impact overall proteostasis independently from its canonical function in regulating circadian rhythm, via protecting against proteome imbalance and proteotoxic stress as recently demonstrated (Wong et al., 2022).

The conservation of ∼12-h gene programs and functionality among Cnidarian, nematode, insects, and mammals argues for an ancient evolutionary origin of ∼12-h oscillator. Since all life originated from the ocean and all in-land animals had to experience tidal environment during ocean-to-land transition in the early era of evolution, it is reasonable to hypothesize that mammalian 12-h oscillator evolved from the circatidal clock of coastal and estuarine animals (Zhu et al., 2017; Zhu et al., 2018; Pan et al., 2020)*.* Although the mechanisms underlying the marine circatidal rhythms remain an open field of research, more and more studies favor the circatidal clock hypothesis (Barford, 2013). For instance, in the crustacean *E. pulchra,* the 12-h circatidal clock is dissociated from the circadian timekeeping system (Zhang et al., 2013). In circatidal mangrove crickets, 12-h rhythms persist in constant darkness, even after the removal of the optic lobe. Furthermore, these 12-h rhythms are intact even when expression of components of the circadian TTFL such as *Clock* or *per* are reduced through siRNA-mediated knockdown (Takekata et al., 2012; Takekata et al., 2014a; Takekata et al., 2014b). What adaptive advantage might 12-h rhythms of gene expression confer? We reason that the ancient ∼12-h rhythms that evolved in marine species in response to tidal cues have been coopted by mammals as an adaptive response to accommodate physiological transitions related to feeding, physical activity, and sleep that are temporally concentrated at dawn and dusk.

Last but not the least, while our study provides strong support for the ∼12-h oscillator hypothesis, we are not ruling out the other two possibilities, namely, that some ∼12-h rhythms may not be cell-autonomous and can be controlled by a combination of the circadian clock and environmental cues, or that they can be regulated by two anti-phase circadian transcriptional factors in a cell-autonomous manner. Future studies using additional *in vivo* and *in vitro* models of circadian deficiency are needed to fully understand the complex relationship between ultradian and circadian rhythms (Asher and Zhu, 2022).

## Materials and methods

### Identification of the oscillating transcriptome

For BMAL1 knockout or WT mice RNA-seq data (GSE171975), raw counts were first normalized to total sequencing depth to get counts per millions (CPM) values, which were used for cycling transcripts identification by either the eigenvalue/pencil or RAIN methods. For the eigenvalue/pencil method (Zhu et al., 2017; Antoulas et al., 2018), the mean expression at each time point were calculated and used as input. A maximum of two oscillations were identified for each gene. Criterion for ∼12-h genes: period between 10 h and 13-h, decay rate between 0.8 and 1.2. The relative amplitude was calculated by dividing the amplitude by the mean expression value for each gene. To determine FDR, we used a permutation-based method that randomly shuffles the time label of gene expression data and subjected each permutation dataset to the eigenvalue/pencil method applied with the same criterion (Rey et al., 2018). These permutation tests were run 5,000 times, and FDR was estimated by taking the ratio between the mean number of rhythmic profiles identified in the permutated samples (false positives) and the number of rhythmic profiles identified in the original data. Analyses were performed in MatlabR 2017A. RAIN analysis was performed as previously described in Bioconductor (3.4) (http://www.bioconductor.org/packages/release/bioc/html/rain.html) (Thaben and Westermark, 2014). FDR (also known as q value) was calculated using the Benjamini-Hochberg procedure.

For RNA-seq in S2 cells (GSE102495), FPKM values reported in the original study were used for cycling transcripts identification. Eigenvalue/pencil analysis was performed as previously described except for that genes with periods between 11 and 14-h are defined as ∼12-h genes. For RAIN analysis, the FPKM data was first subject to polynomial detrend (*n* = 2). For both datasets, heat maps were generated with Gene Cluster 3.0 and TreeView 3.0 alpha 3.0 using either Z score or log2 (fold change over mean) values.

### Gene ontology analysis

DAVID (Version 2021) (Huang da et al., 2009) (https://david.ncifcrf.gov) was used to perform Gene Ontology analyses. Briefly, gene names were first converted to DAVID-recognizable IDs using Gene Accession Conversion Tool. The updated gene list was then subject to GO analysis using either all *Mus musculus* (for mice) or *Drosophila melanogaster* (for S2 cells) genes or hepatically expressed genes (for mice) or all genes expressed in S2 cells as background and with Functional Annotation Chart function. GO_BP_DIRECT was used as GO categories. Only GO terms with a *p*-value less than 0.05 were included for further analysis.

### Motif analysis

Motif analysis was performed with the SeqPos motif tool (version 0.590) embedded in Galaxy Cistrome using all motifs within the mouse reference genome mm9 as background. Genome region spanning 0.5 kb upstream and 05 kb downstream of transcription start were used as input.

### Statistical analysis

Data were analyzed and presented with GraphPad Prism software. Plots show individual data points and bars at the mean and ± the standard error of the mean (SEM). One-tailed t-tests were used to compare means between groups, with significance set at *p* < 0.05. For representative temporal transcritptome data, the curves connecting individual data points were fitted with Cubic spline function in Graphpad. For all period-identification analysis, only the raw data points (but not the fitted Cubic curves) were used.

## Data Availability

Publicly available datasets were analyzed in this study. This data can be found here: GSE171975 and GSE102495.

## Supplementary material

Supplementary Figure S1 Prevalent ∼12-h rhythms of gene expression in the liver of BMAL1 knockout mice. **(A,B)** Heatmap of 12-h rhythms of gene expression uncovered by RAIN with FDR cut-off of 0.1 **(A)** or 0.15 **(B)**. **(C)** Expression of six genes with the lowest q values uncovered by RAIN in BMAL1 knockout mice. Period identified by the eigenvalue/pencil method, and q value (FDR) for having 12-h rhythms of gene expression via the RAIN method were shown for each gene. **(D)** GO analysis showing enriched biological pathways of ∼12-h genes revealed by different methods using hepatically expressed genes as background.

Supplementary Figure S2 Preservation of ∼12-h gene programs and functionality in wild-type and BMAL1 knockout mice. **(A)** Representative ∼12-h genes expression in both wild-type and BMAL1 knockout mice. Period identified by the eigenvalue/pencil method, and q value (FDR) for having 12-h rhythms of gene expression via the RAIN method were shown for each gene in each genotype. **(B)** GO analysis showing enriched biological pathways of ∼12-h genes found in wild-type or in both wild-type and BMAL1 knockout mice using all hepatically expressed genes as background.

Supplementary Figure S3 Distinct functionality of shared and BMAL1 knockout-specific ∼12-h rhythms of gene expression. **(A)** Distribution of periods of wild-type-specific ∼12-h genes in BMAL1 knockout mice and BMAL1 knockout mice-specific ∼12-h genes in wild-type mice. **(B,C)** GO analysis of different ∼12-h genes with all mice **(B)** or hepatically expressed **(C)** genes as background.

Supplementary Figure S4 Many hepatic genes are under dual circadian clock and 12-h oscillator control. **(A)** GO analysis showing enriched biological pathways of common ∼12-h (between wild-type and BMAL1 knockout mice) and circadian (between wild-type and XBP1 LKO mice) using all mice genes as background. **(B)** Eigenvalue/pencil deconvolution of *Ppara* and *Ddx1* temporal gene expression profiles in wild-type, BMAL1 knockout and XBP1 LKO mice. Gray line in each graph illustrates simulated temporal gene expression profile by addition of all superimposed oscillations.

Supplementary Figure S5 Convergence of ∼12-h hepatic transcriptome between wild-type and BMAL1 knockout mice. **(A)** Distribution of periods uncovered from all oscillations by the eigenvalue/pencil method from wild-type and BMAL1 knockout mice. **(B)** Heat map of common ∼12-h rhythms of gene expression uncovered by the eigenvalue/pencil method between wild-type and BMAL1 knockout mice. **(C,D)** Period **(C)** and relative amplitude **(D)** of ∼12-h oscillations for 749 genes in wild-type and BMAL1 knockout mice. **(E)** Scatter plot comparing the phases of 749 ∼12-h oscillations in wild-type and BMAL1 knockout mice. **(F)** GO analysis showing enriched biological pathways of common ∼12-h genes using all mice genes or hepatically expressed genes as background. **(G)** Motif analysis of promoter regions of common ∼12-h rhythm genes identified in wild-type and BMAL1 knockout mice. **(H)** Expression of *Atf6b* in wild-type and BMAL1 knockout mice liver.

Supplementary Figure S6 ∼12-h transcriptome in S2 cells is enriched in protein and RNA metabolism. GO analysis of ∼12-h and circadian rhythms uncovered by different methods/FDR cut-off in S2 cells using all genes expressed in S2 cells as background.

Supplementary Table S1 Eigenvalue/pencil deconvolution of temporal gene expression profile of BMAL1 knockout mice.

Supplementary Table S2 RAIN analysis of temporal gene expression profile of BMAL1 knockout mice.

Supplementary Table S3 Eigenvalue/pencil deconvolution of temporal gene expression profile of 141 genes under dual clocks control in wild-type, BMAL1 knockout and XBP1 LKO mice.

Supplementary Table S4 Eigenvalue/pencil deconvolution of temporal gene expression profile of S2 cell.

Supplementary Table S5 RAIN analysis of temporal gene expression profile of S2 cell.
