# Supplementary figures and images for: Preservation of ∼12-h ultradian rhythms of gene expression of mRNA and protein metabolism in the absence of canonical circadian clock
Source: Front Physiol. 2023 May 30;14:1195001. doi: 10.3389/fphys.2023.1195001 (PMC10267751; doi:10.3389/fphys.2023.1195001)

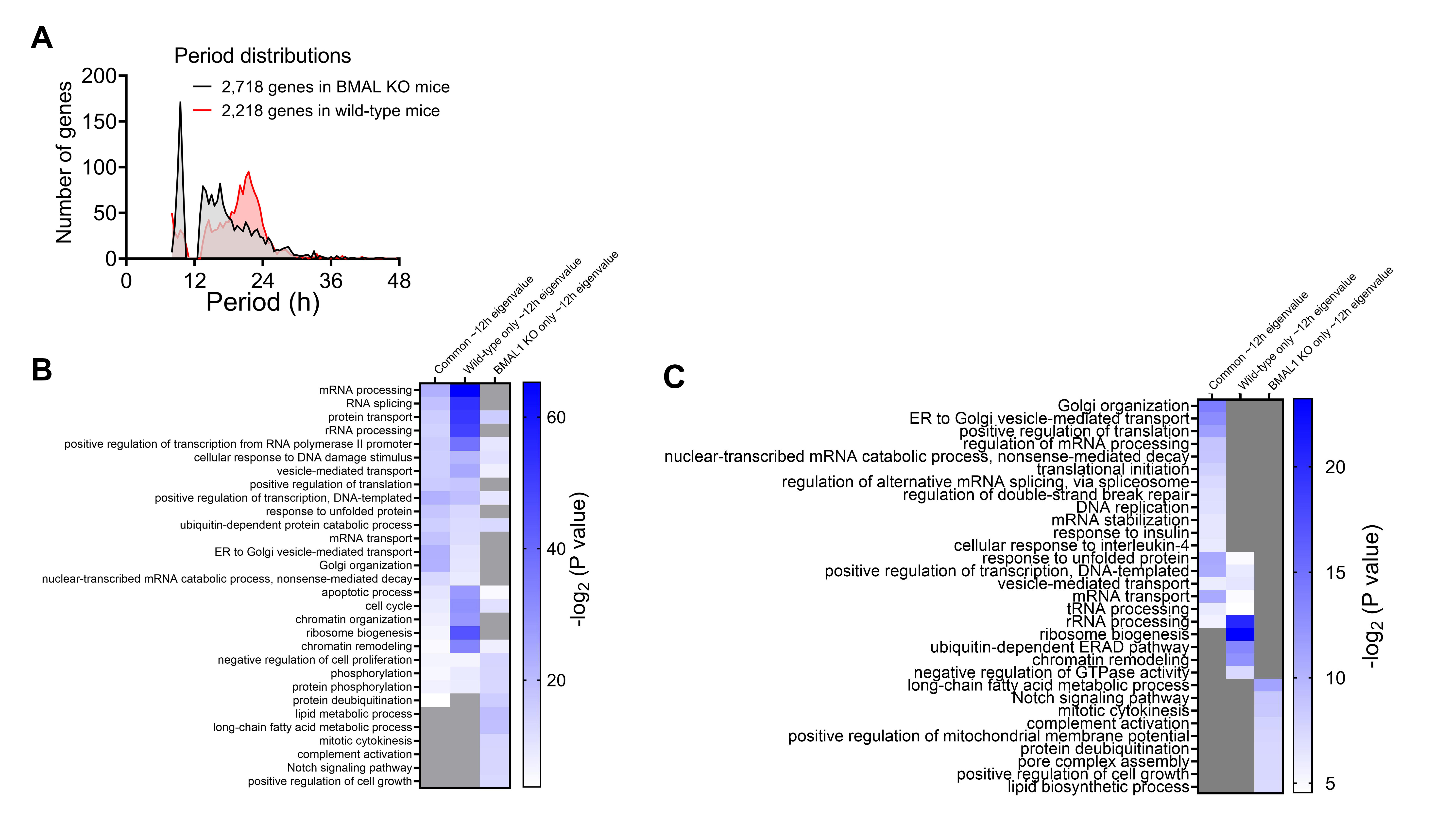

Supplement: Supplementary file 2 [file Image3.JPEG]

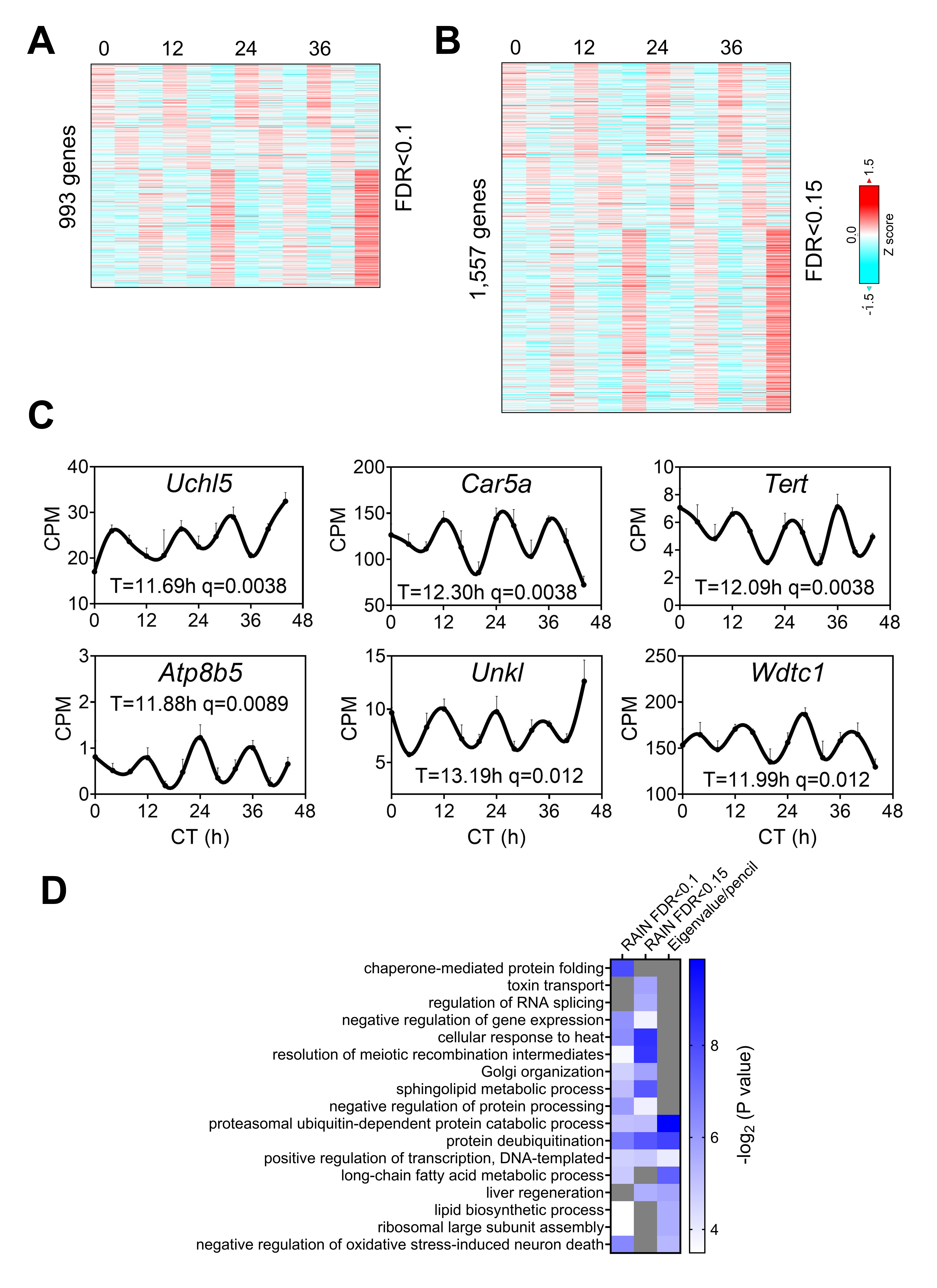

Supplement: Supplementary file 4 [file Image1.JPEG]

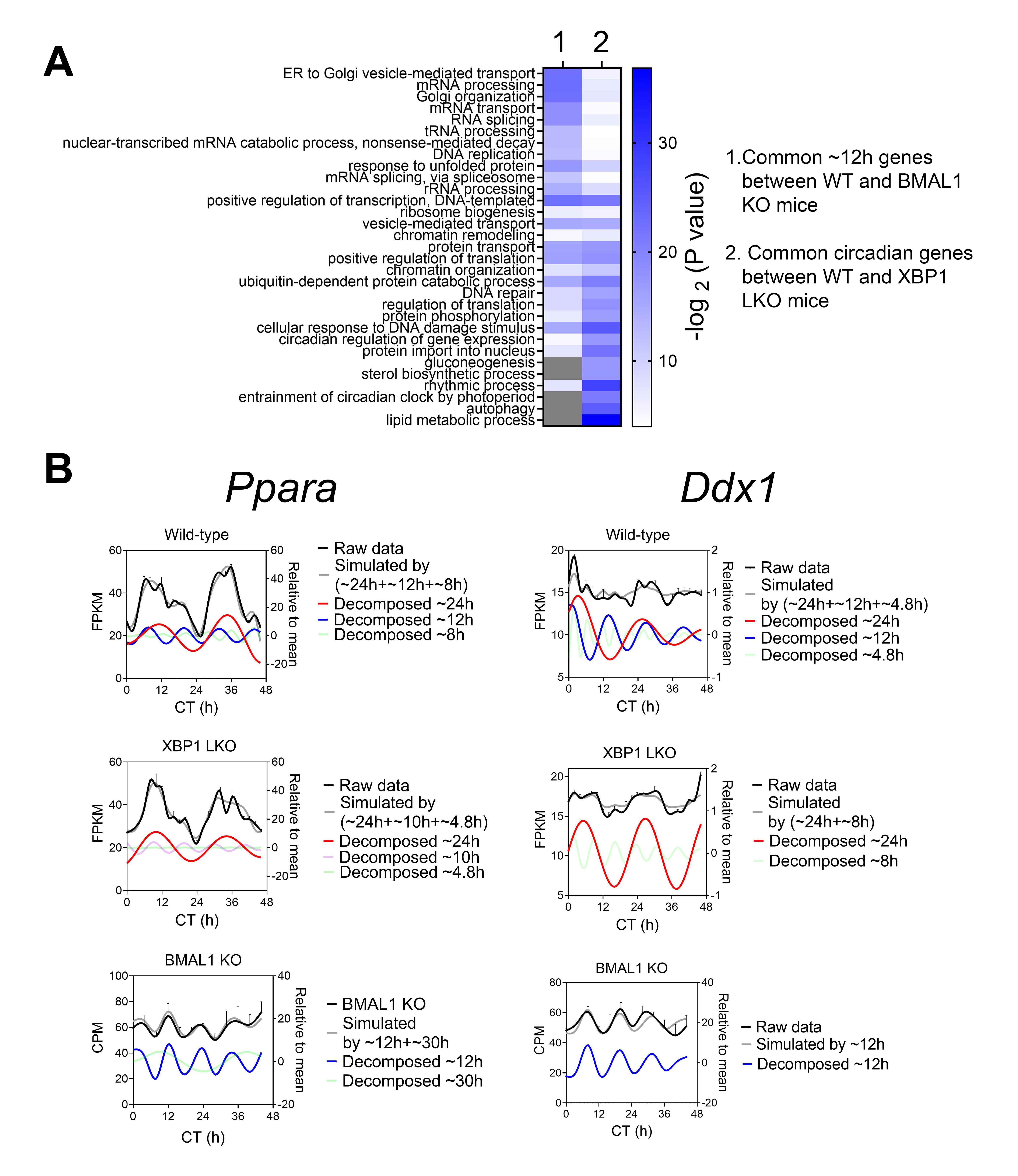

Supplement: Supplementary file 5 [file Image4.JPEG]

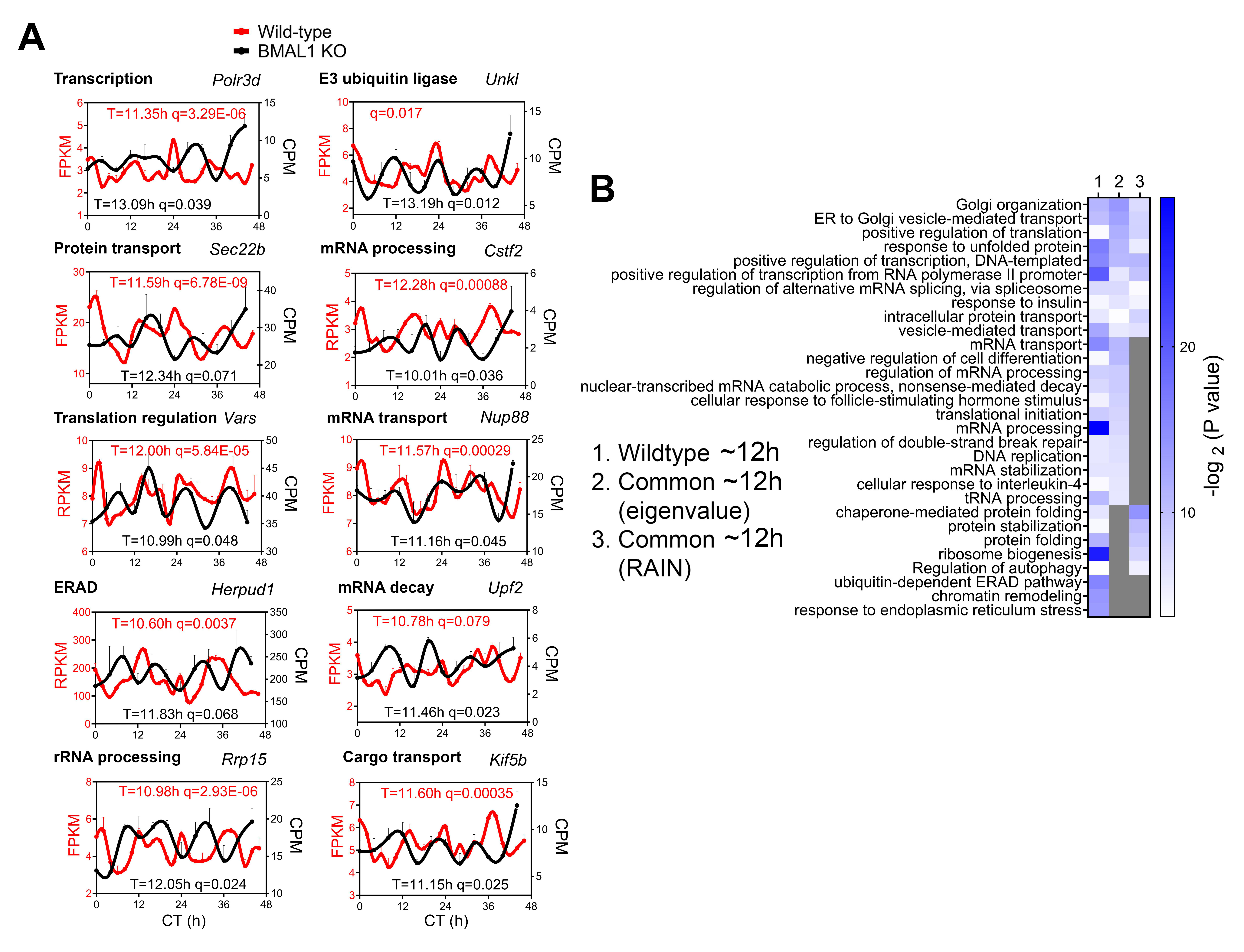

Supplement: Supplementary file 6 [file Image2.JPEG]

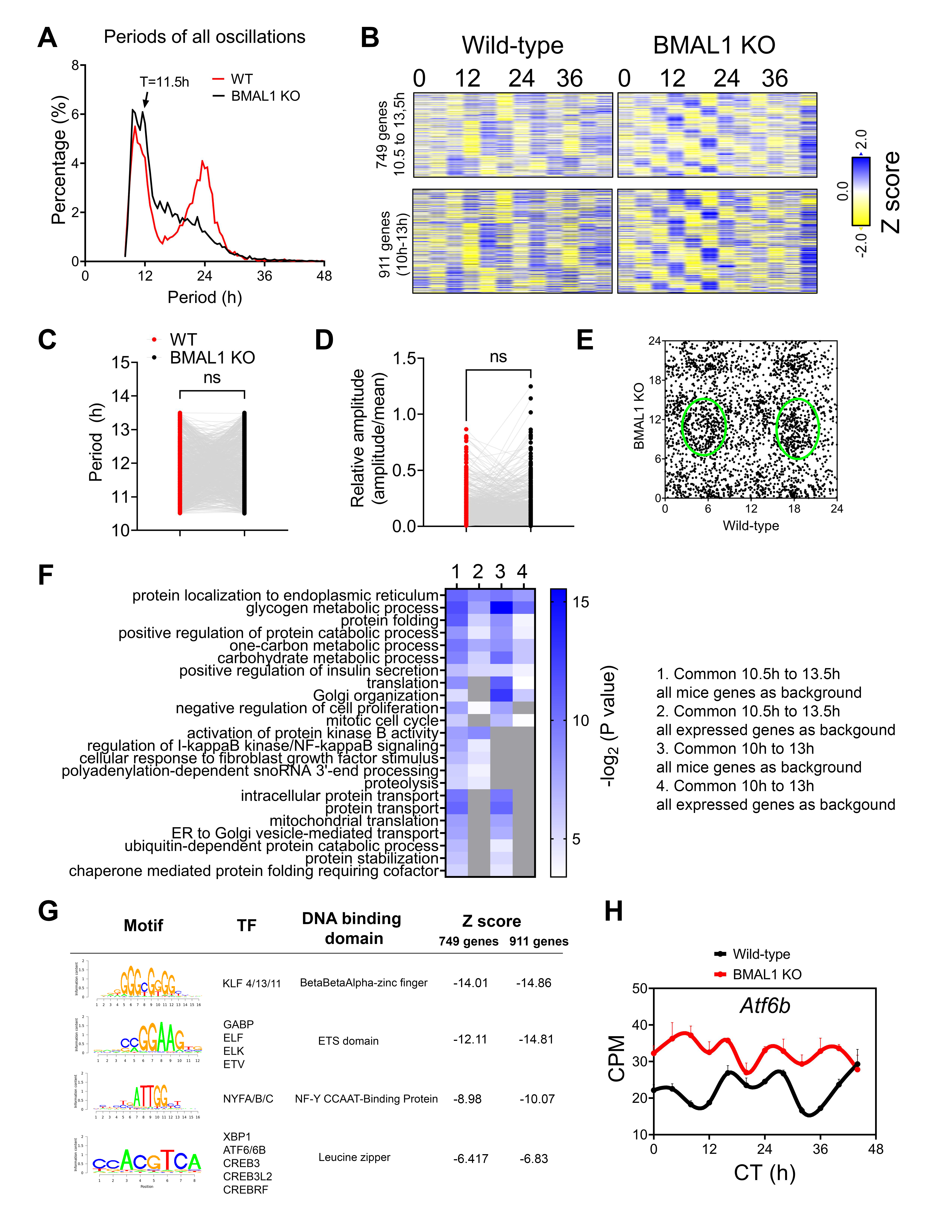

Supplement: Supplementary file 7 [file Image5.JPEG]

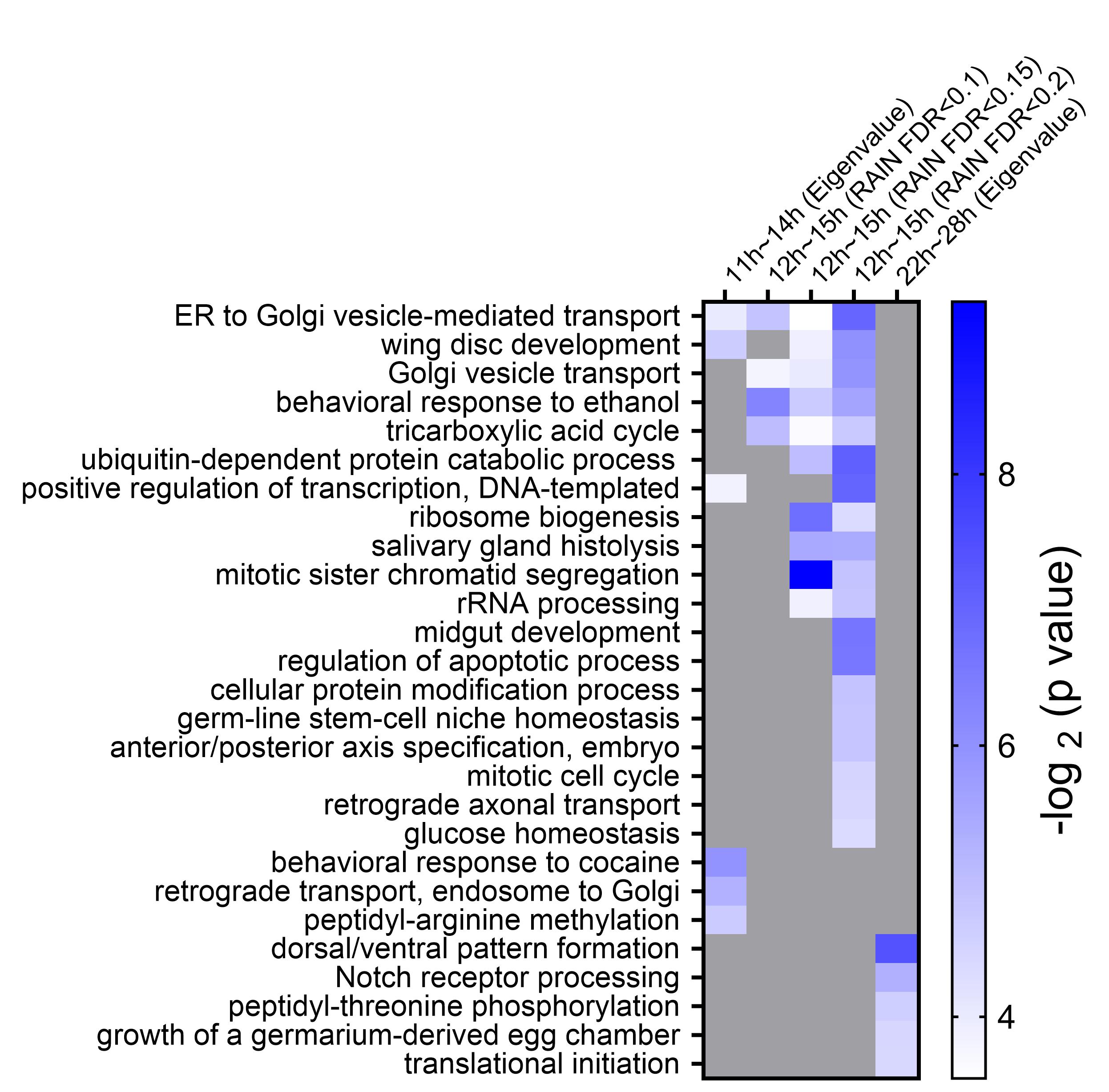

Supplement: Supplementary file 11 [file Image6.JPEG]
